# Prevalence of porcine circovirus-like agent P1 in Jiangsu, China

**DOI:** 10.1186/1743-422X-8-543

**Published:** 2011-12-15

**Authors:** Libin Wen, Kongwang He, Hanchun Yang, Zhengyu Yu, Aihua Mao, Shulin Zhong, Yanxiu Ni, Xuehan Zhang, Bin Li, Xiaomin Wang, Junming Zhou, Rongli Guo, Lixin Lv, Jieyuan Jiang

**Affiliations:** 1 Institute of Veterinary Medicine, Jiangsu Academy of Agricultural Sciences·Key Laboratory of Veterinary Biological Engineering and Technology, Ministry of Agriculture·National Center for Engineering Research of Veterinary Bio-products, Nanjing210014, China; 2 College of Veterinary Medicine, China Agricultural University, Beijing 100094, China

**Keywords:** Porcine circovirus type 2-like agent P1, Prevalence, PCR, China

## Abstract

Recently, we identified a novel porcine circovirus type 2-like agent P1 isolate from swine. The present study represents the first survey of P1 prevalence in swine herds from Jiangsu, China, by using PCR targeting the complete genome of P1. Prevalences of 50% and 19% were found among 6 herds and 248 animals, respectively. The results indicate a high prevalence of P1 in China pig populations.

## Introduction

Porcine circovirus (PCV), belonging to the family *Circoviridae*, is a small non-enveloped virus with a circular, single-stranded DNA genome. To date, two types of porcine circoviruses have been recognized: PCV1 and PCV2. PCV1 was originally isolated from the porcine kidney cell line PK15 [1] and did not induce a disease in swine [2], while PCV2 has recently been identified as the causal agent of postweaning multisystemic wasting syndrome (PMWS) [3,4]. The major clinical signs of PMWS are progressive wasting and growth retardation, pallor of the skin, and occasional icterus [5,6].

PCV2-like agent (designated P1) was discovered by accident in 2007 from the serum of the porcine with PMWS by PCR assay. Briefly, a pair of primers was designed according to the nucleotide sequence of PCV2 ORF2 (AF381175): forward primer F1 (5'-ACGGATATTGTAGTCCTGGT-3') and reverse primer R1 (5'-CAAGGCTACCACAGTCAGAA-3'). This pair of primers amplifies a 472-bp DNA fragment of PCV2. Unexpectedly, a truncated amplicon with 1 nucleotide deletions was generated by DNA sequencing, which will result in frameshift mutation and a premature termination codon of ORF2 protein synthesis. According to the results above, we suspected that the existence of the agent related with ORF2 sequences of PCV2 might be possible. Thus, inverse PCR was carried out with a pairs of primers based on DNA sequence of the amplicon, F2: 5'-TGTAGACCACGTAGGCCTCGG-3', R2: 5'-GGTTTGTATCCTCAGCCAAAG-3'. Then, the sequences of PCR generated fragments were assembled into consecutive sequence by using DNAMAN software (Version 5.2.2, Lynnon Biosoft, 1994).

Using inverse PCR bands should only be amplified if the agent genome is circular. The exception to this would be if there was non-specific binding of one of the primers. This is very unlikely when fragments have been amplified with two different primer sets but we still designed another pair of primers based on the sequences assembled to amplify the agent complete genome containing overlapping regions to verify.

**Figure 1:**
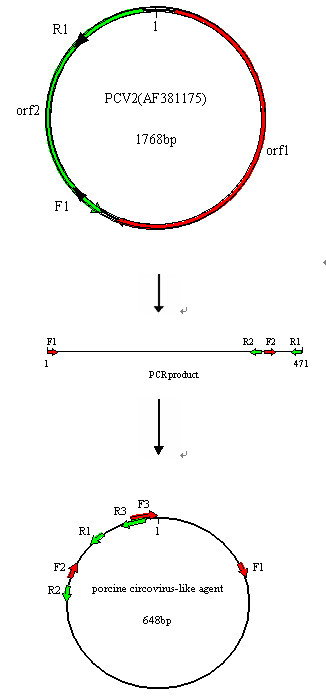
**Schematic diagram of the PCR process to obtain porcine circovirus-like agent P1**.

F3: 5-TTAAAGACCCCCCACTTAAACCCTAAATGA-3', and R3: 5'-AGTGGGGGGTCTTTAAGATTAAATTCTCTG-3'. (Figure 1).

The novel PCV2-like agent (P1) was determined with a circle DNA genome of 648 nucleotides. The phylogenetic analysis showed that the virus P1 closely related to the known virus is PCV. It showed 98.42% of nucleotide homology with PCV2 BF isolate. The partial sequence data of P1 have been submitted to GenBank (EF514716), except 5'terminal 22 nt-------"ggatccactagtaacggccgcc". P1 genome has three possible open reading frames (ORF), capable of encoding amino acids of 12.5 kD (ORF1), 3.0 kD (ORF2) and 3.9 kD (ORF3), respectively [7]. P1 has high homologous sequences of ORF2 of PCV2 and ORF3 of P2, another novel porcine circovirus-like agent [8]. The genome structure suggests that P1 and P2 might belong to a new virus family. So far, Very little is known about the epidemiology of P1. Therefore, the main objective of the present work was to determine the prevalence of P1.

## Materials and methods

**Figure 2:**
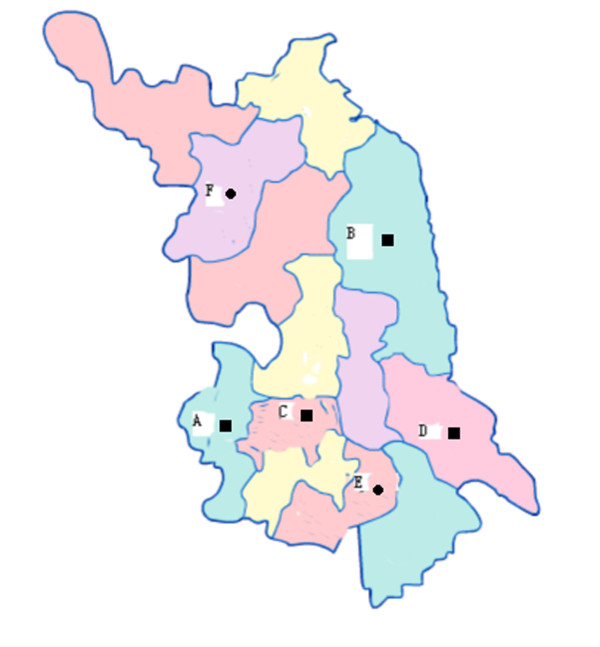
**Geographic distribution of the pig herds involved in this study**. Black sphere(●) represents samples collected in 2010 and black square(■) represents samples collected in 2009. English alphabet represents the herds in different parts of the Jiangsu; A in Nanjing, B in Yancheng, C in Zhenjiang, D in Nantong, E in Wuxi, and F in Suqian.

Initially, 248 pig sera corresponding to 6 non-related, different size herds sampled during years 2009 and 2010 were used for this study. Specifically, these consisted of 4 herds (A, B, C, D) in 2009, and 2 herd (E, F) in 2010, and the herds were located in the north western (Su qian), eastern (yancheng and Nantong), southern and central (Nanjing, zhenjiang, and wuxi) parts of Jiangsu province (Figure 2). The pigs were between 10 and 120 days of age and submitted to clinic examination because of different clinicopathological conditions, including wasting, respiratory distress, diarrhoea, and ochrodermia. Sera samples were stored at -20°C for viral DNA extraction and PCR analysis.

For the viral DNA extraction, 300 μL of cell lysis buffer (20 mM EDTA, 0.5% sodium dodecyl sulphate (SDS) and 200 μg/ml proteinase K) was mixed with the 300 μL serum. Thoroughly vortexed and incubated at 50°C for 2 h. Following incubation, DNA was extracted by conventional phenol -chloroform methods.

The specific P1 DNA was determined by using one-step PCR assay targeting complete nucleotide sequence of viral DNA-genome. The primers used for PCR reactions were previously designed [7]. Briefly, the PCR was performed in a 25 μL final volume containing 2.5 μL of viral template DNA, 10 pmol of forward primer F3 and reverse primer R3, 2.5 mM dNTPs and 0.75 U DNA Polymerase. The amplification was performed using a PCR thermal cycler (TaKaRa) and was initiated by heating for 5 min at 94°C, followed by 40 cycles denaturation at 94°C for 45 s, annealing at 58°C for 45 s, and elongation at 72°C for 45 s. The reaction ended with a final extension step at 72°C for 10 min. Finally, 15 μL of PCR product was run on 1.5% TAE-agarose gel stained with ethidium bromide (0.5 μg/mL). Positive DNA fragments of specific size (approximately 660 bp) were excised from the agarose gel and purified by using AxyPrep™ DNA Gel Extraction kit (Hangzhou, China), and finally TA-cloned into vector pMD-18 T (TaKaRa) for sequencing. Three or 4 PCR positive samples for each pig herd were selected for sequence identification.

## Results

Of the 248 serum specimens obtained from swine, 47(19%) were P1-positive by PCR assay, whilst 3(50%) were positive for P1 among 6 farms. Prevalence values vary from 0% to 50% in pig herds. The prevalence results of P1 in six different geographical regions in Jiangsu are shown in Table 1. P1 infection rate of the pigs differed with their ages as Table 2. There was significant difference on the prevalence of different age stage, being 50.0% (13/26) (10-30 days), 11.1% (3/27) (40-60 days), 22.2% (6/27) (70-90 days) and 16.0% (4/25) (100-120 days), respectively. 10-30 days has the highest number of infected pigs in herd F infected with P1.

**Table 1:** Number of positive pigs by P1 PCR in different herds and sera of pigs with PMWS

Pig group	No. tested pigs	No. PCR P1 positive pigs (%)
A	20	10 (50.0)

B	40	11 (27.5)

C	20	0(0)

D	15	0(0)

E	48	0(0)

F	105	26(24.8)

**Table 2:** The representative ages and the number of pigs in herd F tested for P1 in serum samples by PCR

Age of pigs(days)	No. tested pigs	No. P1-affected
10-30	26	13

40-60	27	3

70-90	27	6

100-120	25	4

Some of the PCR products were cloned and sequenced. All of the P1 strains was 648 bp in size and shared more than 97.2% nucleotide homology with the isolated P1 strain submitted to GenBank (EF514716).

## Discussion

PMWS, a new emerging and multifactorial disease in swine, was first recognized in North America in 1991 [9]. Since then, this disease has been diagnosed globally and devastated almost every pig-producing area of the world [10]. A role of PCV2 in the etiology of PMWS was first observed in Canada in 1991, and described in the late 1990s. Although PCV2 infection has been associated with PMWS, the role of PCV2 infection in the pathogenesis of PMWS is uncertain. To date, PCV-2 infection is regarded as a necessity in PMWS cases, other cofactors are necessary to evoke disease. PMWS-affected pigs exhibit a wide spectrum of concomitant infections with PCV2, such as porcine parvovirus [11,12], porcine reproductive and respiratory syndrome virus (PRRSV) [13,14], TTV [15] and Mycoplasma hyopneumoniae [16], all may enhance PCV2-associated lesions and develop whole complex clinical symptom of PMWS under experimental and field conditions [17].

The difficulties in reproducing the full spectrum of clinical signs and lesions associated with PMWS using PCV2 alone have resulted in a hypothesis that PMWS may be triggered by an unknown pathogen, popularly termed 'agent X', Of course, no such novel agent has been identified until now [18]. The finding of novel viral agents in the PMWS disease is challenging since there still are many open questions concerning the aetiology of the syndrome. From all the studied animals in the present study, 3 were co-infected with both PCV2 and P1, 10 were positive for PCV2 only (data not shown). Therefore, further studies needs to be conducted in order to see if the presence of the novel agent P1 described here, contributes to the development of PMWS solely and/or in synergy with PCV-2.

PCR was proved to be a useful tool for detecting PCV2 in clinical specimens because of its speed, specificity, and sensitivity. But challenges encountered by its specificity as to P1 occurrence, most of PCR assay reported previously would target the ORF2 region of the PCV2 genome, which shared high similarity nucleotide sequence with ORF1 of P1. It is vital to determine whether those PCR amplification fragments were produced by PCV2 or by P1. The PCR assay described here should provide the ability to identify and differentiate between PCV2 and P1 and screen pigs for P1 infection. To our knowledge, this is the first report on epidemiology of P1 in China. Of course, to obtain the complete genome sequence of P1 and aid in further analysis of genomic features of P1, we used the pairs of primers F3 and R3 including an overlapping region in this study. Rather than amplify the entire 648 nt of P1, designing some primers amplifying smaller products may improve the sensitivity of the assay and be more suitable for epidemiological investigation of P1.

In a previous study, 1.8% (5/278) of studied swine sera from China was shown to be P1-positive by a PCR detecting [7]. The difference in the prevalence of P1 (19%, 47/248) obtained in our present study indicate that the P1 infection has became popular in pig herds. Of course, very low copies of P1 DNA were also detected in some sera samples, the biological importance of the virus remains to be elucidated.

In summary, the present work contributes to the understanding of P1 epidemiology, including the establishment of PCR assay described here. Taking into account the present results, it seems evident that P1 infection is already widespread in Jiangsu and those 10-30 days old pigs seem more likely to be infected with P1. Further studies should be investigated in larger scale data in order to determine the biological importance of P1.

## Competing interests

The authors declare that they have no competing interests.

## Authors' contributions

LW contributed in the study design of the study and drafted manuscript. KH and HY were responsible for the planning of the study. ZY, AM, SZ, YN, XZ, and BL participated in clinical samples collection. XW and JZ participated in results analysis. RG and LL performed the work. JJ participated in revising the manuscript. All the authors read and approved the final manuscript.
